# Impact of age at appendectomy on development of type 2 diabetes: A population-based cohort study

**DOI:** 10.1371/journal.pone.0205502

**Published:** 2018-10-16

**Authors:** Yang-Ming Lee, Chew-Teng Kor, Diko Zhou, Hsueh-Chou Lai, Chia-Chu Chang, Wen-Lung Ma

**Affiliations:** 1 Graduate Institution of Clinical Medical Science, and Graduate Institute of Biomedical Sciences, China Medical University, Taichung, Taiwan; 2 Internal Medicine Research Center, Changhua Christian Hospital, Changhua, Taiwan; 3 BioActive Lipid Research Center (BALRC), Department of Surgery, BenQ Medical Center, Suzhou, Jiangsu Province, China; 4 Sex Hormone Research Center, and Department of Gastroenterology, China Medical University Hospital, Taichung, Taiwan; 5 School of Medicine, Chung Shan Medical University, Taichung, Taiwan; National Yang-Ming University, TAIWAN

## Abstract

**Aim:**

Diabetes is a complex metabolic disease characterized by chronic low-grade inflammation in which genetic and environmental factors are involved. Growing evidence implicates that alterations of the gut microbiota potentially contribute to the emergence of metabolic diseases. The human appendix has more recently been recognized as a microbial reservoir for repopulating the gastrointestinal tract and an important part of the immune system. Thus, appendectomy may influence microbial ecology and immune function. This study investigated the association between appendectomy and type 2 diabetes risk.

**Methods:**

We analyzed a cohort of 10954 patients who underwent appendectomy between 1998 and 2013 based on the Taiwan National Health Insurance Program database. A comparison cohort of 43815 persons without appendectomy was selected randomly and matched by sex, age, comorbidities, and index year. To ensure reliability of the results, a sensitivity analysis using a propensity score–matched study was performed. We observed the subsequent development of type 2 diabetes in both cohorts.

**Results:**

Although the overall incidence of type 2 diabetes in the appendectomy patients was 7.9% higher than that in the non-appendectomy patients, it was not statistically significant (95% confidence interval [CI], 0.997–1.168) after the adjustment of confounding factors. Multivariate regression analysis revealed that the adjusted hazard ratio (HR) of type 2 diabetes was 1.347 for appendectomy patients < 30 years of age (95% CI, 1.009–1.798) compared to non-appendectomy patients. The incidence of type 2 diabetes was higher within 3 years of post-appendectomy follow-up than for non-appendectomy patients (HR, 2.017; 95% CI, 1.07–3.802). Age impacted the association between appendectomy and type 2 diabetes risk (P_interaction_ = 0.002); in contrast, sex did not affect the association between appendectomy and type 2 diabetes risk (P_interaction_ = 0.88).

**Conclusions:**

Our study results suggest that appendectomy increases type 2 diabetes risk, particularly when performed prior to middle age.

## Introduction

Diabetes is one of the most serious public health issues worldwide due to its rapidly increasing incidence and tremendous amount of various vascular complications. According to the International Diabetes Federation, in 2015, the 415 million individuals had diabetes, a figure that is estimated to increase to 642 million by 2040 [1] Diabetes is a complicated metabolic disease characterized by chronic inflammation [2, 3] that involves genetic and environmental factors. Insulin resistance, the core defect of metabolic diseases, is linked to low-grade inflammation; however, its detailed molecular mechanism remains to be determined. Recent studies have suggested that the gut microbiota play a fundamental role in metabolic disease development by modifying energy homeostasis [4–8]. Studies from animal models have shown that alterations in the gut microbiota contribute to the development of metabolic diseases by increasing endotoxemia-induced inflammation [9]. Furthermore, Cox et al. demonstrated that altering the intestinal microbiota in early life using low-dose penicillin switched the metabolic phenotype [10]. Ridaura et al. demonstrated that the microbiota derived from discordant obese twins affected murine metabolism [11].

Despite long being considered an evolutionary vestigial organ, the human appendix has more recently been recognized as an important part of the immune system. Furthermore, the human appendix may function as a “safe house” to preserve and protect commensal bacteria in the gastrointestinal tract via immune-mediated biofilm formation [12–15]. Consequently, appendectomy may change the gut microbiota composition and immune function, subsequently promoting the emergence of various inflammatory diseases, including colon cancer, Crohn’s disease, and rheumatoid arthritis [16–21]. This study aimed to investigate whether appendectomy increases the risk of type 2 diabetes (DM).

**Informed consent statement.** Although written informed consent was not provided by the participants for the use of their medical records in this study, the patient records were de-identified and anonymized prior to the investigation. The work was approved after a full ethical review by the Institutional Review Board (IRB) of the Changhua Christian Hospital (approval number 171210), and the IRB waived the need for consent.

## Material and methods

### Study population

**Fig 1:**
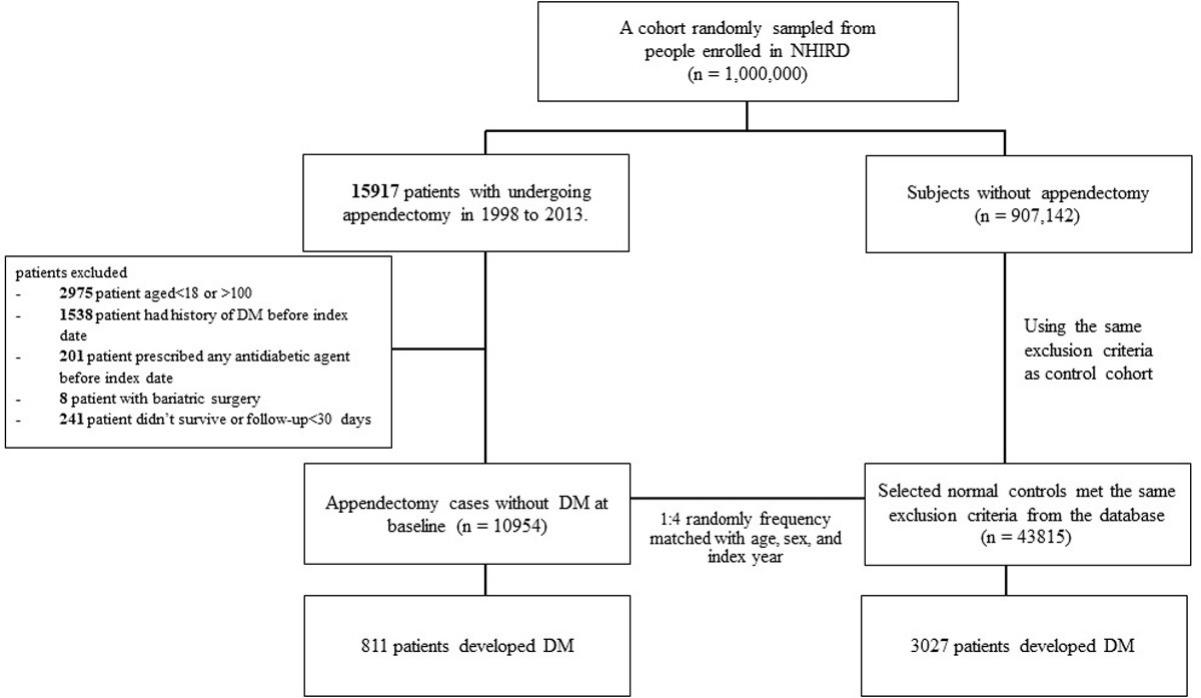
Flow chart of the study subjects illustrating how we analyzed a cohort of 10954 patients who underwent appendectomy between 1998 and 2013 based on Taiwan National Health Insurance Program data. A comparison cohort of 43815 persons without appendectomy was randomly selected and frequency matched by sex, age, comorbidities, and index year. We observed the subsequent development of diabetes in both cohorts.

We used a one-million-person random sample from people enrolled in the Taiwan National Health Insurance Program (NHIP). Started in 1995, the program includes 99% of the 23-million-plus people who live in Taiwan. This database from the NHIP is managed and established for investigators. With the hospitalization database from the Taiwan NIHP, the appendectomy group was determined as patients aged 20–84 years undergoing appendectomy from 1998 to 2013 (the International Classification of Diseases [ICD] 9^th^ Revision, ICD-9 procedure codes 47.0 and 47.1). We identified a study group of 15917 patients who underwent appendectomy between 1998 and 2013. We excluded 2975 persons aged <18 or >100 years, 1538 persons with a history of DM history before the index date, 201 patients with any antidiabetic agent history before the index date, eight patients who underwent bariatric surgery, and 241 patients who did not survive or were followed up for < 30 days. The remaining 10954 patients were included in the study as the appendectomy group. The index year was determined as the year the appendectomy was performed. We established a random frequency-matched study cohort for assessing the association between appendectomy and DM. For each identified appendectomy patient, four control groups were frequency matched by age, sex, and index year. A total of 10954 appendectomy patients and 43815 controls were included in the study cohort (Fig 1). We also performed a propensity score-matched sensitivity analysis to ensure the reliability of our results. Propensity scores were calculated using multivariate logistic regression to predict the probability of DM occurrence to balance the covariate distribution in the appendectomy and control groups. Based on the propensity score, 1:4 propensity score matching was performed in the sensitivity analysis.

All comorbidities were diagnosed with ICD-9 codes. The following comorbidities potentially related to DM included: hypertension (ICD-9 401–405), hyperlipidemia (ICD-9 272), gout (ICD-9 274), polycystic ovaries (ICD-9 256.4), cerebrovascular disease (ICD-9 430–438), renal disease (ICD-9 580–589), ischemic heart disease (CAD; ICD-9 410–414), chronic obstructive pulmonary disease (COPD; ICD-9 416.8, 416.9, 490–505, 506.4, 508.1, and 508), gestational diabetes (GDM) (ICD-9 648.0, 648.8), depression (ICD-9 296.2, 296.3, 300.4, and 311), obesity (ICD-9 278, 783.1, and V77.8), chronic pancreatitis (ICD-9 577.1), hepatitis B infection (HBV; ICD-9 070.20, 070.21, 070.22, 070.23, 070.30, 070.31, 070.32, 070.33, and V02.61), and hepatitis C infection (HCV; ICD-9 070.41, 070.44, 070.51, 070.54, 070.7, and V02.62).

The major outcome was a new diagnosis of DM (ICD-9 250.0–250.9) based on at least three outpatient diagnoses or one hospital discharge diagnosis during the follow-up period. All study subjects were followed until they were diagnosed with DM at the end of 2013.

### Statistics

Demographic and clinical characteristics in the appendectomy and non-appendectomy subjects were presented as proportions and mean ± standard deviation (SD). The standardized differences were used with an absolute value for the standardized difference of >0.1 considered to indicate important imbalances between the two groups for categorical or continuous matching variables, respectively. The incidence of DM was measured as the event number of DM diagnosed during the follow-up year divided by the total follow-up person-years for each subject.

The Cox proportional hazards regression model was applied to measure the hazard ratio (HR) and 95% confidence interval (CI) for the risk of DM in the appendectomy compared to non-appendectomy groups. Multivariate Cox analysis with cause-specific hazard models was used to estimate adjusted hazard ratios (aHRs) adjusted for confounders for appendectomy and other comorbidities including hypertension, hyperlipidemia, gout, polycystic ovaries, depression, cerebrovascular disease, renal disease, CAD, COPD, GDM, obesity, chronic pancreatitis, HBV infection, HCV infection, medications (statins, atypical antipsychotics, HIV drug and corticosteroids for systemic immunosuppressants) and number of visiting clinics.

Uni- and multivariate survival analyses were performed for the incidence and relative risk of DM. A subgroup analysis was used to distinguish between DM risks in the appendectomy and non-appendectomy groups. We then used propensity score-matched analysis to assess the reliability of our results. Propensity scores were calculated using multivariate logistic regression to predict the probability of DM occurrence. The cumulative incidence curves from cause-specific hazard methods for the appendectomy and control groups were also analyzed.

## Results

Of the 15917 patients in the appendectomy group, 10954 patients remained after the exclusion of 2975 patients <18 or >100 years of age, 1538 patients with a history of DM, 201 patients with a prescription history of antidiabetic agents, eight patients who underwent bariatric surgery, and 241 patients who did not survive or completed <30 days of follow-up. There were a total of 43815 persons in the control group.

Comparisons in demographic characteristics are shown in Table 1. The mean ages of the appendectomy and control groups were similar (39.5 years), and each was predominantly composed of males (51.06%). In cohort 1 (random frequency-matched analysis), the appendectomy and control groups were matched by hypertension, hyperlipidemia, gout, polycystic ovaries, depression, GDM, depression, obesity, chronic pancreatitis, HBV and HCV, statins, atypical antipsychotics, HIV drugs, corticosteroids, and immunosuppressants. There was a slight difference in Charlson Comorbidity Index score and number of visiting clinics between the two appendectomy and control groups in cohort 1.

**Table 1:** Comparisons in demographic characteristics, comorbidities medications and clinical outcomes in subjects with and without appendectomy.

	Cohort1 in Main Result (Randomly Frequency matched Cohort)			Cohort 2 in sensitivity analysis (Propensity score matched Cohort)		
	Nonappendectomy	Appendectomy	StD^a^	Nonappendectomy	Appendectomy	StD^a^
Sample size	43815	10954		43815	10954	
Gender						
F	21443(48.94%)	5361(48.94%)	0.000	21252(48.5%)	5361(48.94%)	0.009
M	22372(51.06%)	5593(51.06%)	0.000	22564(51.5%)	5593(51.06%)	0.009
Age stratified						
<20	2270(5.18%)	635(5.8%)	0.027	2596(5.92%)	635(5.8%)	0.0054
20–39	22216(50.7%)	5528(50.47%)	0.005	22438(51.21%)	5528(50.47%)	0.0149
40–59	13818(31.54%)	3401(31.05%)	0.011	13644(31.14%)	3401(31.05%)	0.0020
60–79	4896(11.17%)	1233(11.26%)	0.003	4537(10.35%)	1233(11.26%)	0.0290
> = 80	615(1.4%)	157(1.43%)	0.003	601(1.37%)	157(1.43%)	0.0052
Age, years	39.58±15.7	39.5±15.76	0.005	39.08±15.55	39.5±15.76	0.038
Monthly income, NTD						
<15840	19924(45.47%)	5182(47.31%)	0.037	20719(47.29%)	5182(47.31%)	0.000
15840−25000	13792(31.48%)	3561(32.51%)	0.022	14107(32.2%)	3561(32.51%)	0.007
≥25000	10099(23.05%)	2211(20.18%)	0.070	8990(20.52%)	2211(20.18%)	0.008
Number of visiting clinic	15.24±13.75	16.97±14.85	0.165	16.85±14.78	16.97±14.85	0.012
Charlson’s comorbidity index						
0	32080(73.22%)	6788(61.97%)	0.242	27272(62.24%)	6788(61.97%)	0.006
1–2	9937(22.68%)	3331(30.41%)	0.176	13479(30.76%)	3331(30.41%)	0.008
≥3	1798(4.1%)	835(7.62%)	0.150	3065(7%)	835(7.62%)	0.024
Comorbidity at baseline						
Hypertension	3462(7.9%)	1077(9.83%)	0.068	3896(8.89%)	1077(9.83%)	0.032
Hyperlipidemia	1313(3%)	442(4.04%)	0.056	1355(3.09%)	442(4.04%)	0.051
Gout	938(2.14%)	323(2.95%)	0.051	1088(2.48%)	323(2.95%)	0.029
Polycystic ovaries	104(0.24%)	35(0.32%)	0.016	114(0.26%)	35(0.32%)	0.011
Gestational diabetes	29(0.07%)	19(0.17%)	0.031	47(0.11%)	19(0.17%)	0.018
Depression	440(1%)	143(1.31%)	0.028	489(1.12%)	143(1.31%)	0.017
Obesity	114(0.26%)	40(0.37%)	0.019	101(0.23%)	40(0.37%)	0.025
Chronic pancreatitis	19(0.04%)	11(0.1%)	0.021	31(0.07%)	11(0.1%)	0.010
Hepatitis B infection	429(0.98%)	189(1.73%)	0.065	693(1.58%)	189(1.73%)	0.011
Hepatitis C infection	138(0.31%)	45(0.41%)	0.016	135(0.31%)	45(0.41%)	0.017
Medication at baseline						
Statins	559(1.28%)	196(1.79%)	0.042	649(1.48%)	196(1.79%)	0.024
Atypical Antipsychotics	39(0.09%)	9(0.08%)	0.002	20(0.05%)	9(0.08%)	0.014
HIV-drug	20(0.05%)	10(0.09%)	0.017	36(0.08%)	10(0.09%)	0.003
Corticosteroids for systemic	338(0.77%)	189(1.73%)	0.086	648(1.48%)	189(1.73%)	0.020
Immunosuppressants	69(0.16%)	22(0.2%)	0.009	70(0.16%)	22(0.2%)	0.010
Propensity score	2.92±1.4	3.33±1.41	0.409	0.02±0.01	0.02±0.01	0.000
Outcome						
DM	3027(6.91%)	811(7.4%)	0.019	2819(6.43%)	811(7.4%)	0.0382
death	2660(6.07%)	705(6.44%)	0.015	2560(5.84%)	705(6.44%)	0.0247

^a^ StD, standardized difference of greater than 0.1 is considered important imbalance.

To assess the reliability of cohort 1, a propensity score-matched sensitivity analysis (cohort 2) was conducted, and it showed that the appendectomy and control groups had equal distributions of all demographic characteristics. Overall, 7.4% (n = 811) of patients with appendectomy developed type 2 DM versus 6.91% (n = 3027) of persons without appendectomy in cohort 1. Similar results were shown in cohort 2. Using this data mining strategy, we were able to examine the correlation between appendectomy and the development of type 2 DM. As shown in Table 2, we found the overall incidence of type 2 DM in the appendectomy patients was 7.9% higher than that in the control group, although the difference was not statistically significant (P = 0.06). The incidence of type 2 DM in the appendectomy patients < 30 years of age was 1.453-fold greater than that in the control group (95% CI, 1.093–1.933). The multivariable regression analysis revealed that the adjusted HR of type 2 DM was 1.347 for the appendectomy patients < 30 years (95% CI, 1.009–1.798) compared to controls. Age affected the association between appendectomy and type 2 DM (P_interaction_ = 0.002), whereas sex did not (P_interaction_ = 0.88).

**Table 2:** Incidence and hazard ratios of diabetes mellitus for appendectomy patients compared with non-appendectomy cohort by demographic characteristics and comorbidities.

Variables	Subjects with non-appendectomy				Subjects with appendectomy					Appendectomy vs. Non- appendectomy cohort			P_interaction_
	N	Event	PY^†^	incidence^‡^	N	Event	PY^†^	incidence^‡^	cHR (95% CI)	p-value	aHR^§^ (95% CI)	p-value	
All patients	43815	3027	340573.93	8.89 (8.57,9.2)	10954	811	83432.67	9.72 (9.05,10.39)	1.091 (1.01,1.179)	0.03	1.079 (0.997,1.168)	0.06	
Gender													0.88
Male	22372	1657	170069.30	9.74 (9.27,10.21)	5593	445	41937.96	10.61 (9.63,11.6)	1.091 (0.983,1.211)	0.10	1.058 (0.939,1.192)	0.34	
Female	21443	1370	170504.63	8.03 (7.61,8.46)	5361	366	41494.71	8.82 (7.92,9.72)	1.092 (0.973,1.225)	0.13	1.072 (0.964,1.192)	0.20	
Age, years													**0.002**
** <30**	**14041**	**179**	**119669.38**	**1.5** **(1.28,1.71)**	**3564**	**64**	**29876.48**	**2.14** **(1.62,2.67)**	**1.453** **(1.093,1.933)**	**0.01**	**1.347** **(1.009,1.798)**	**0.04**	
30–40	10445	428	83434.68	5.13 (4.64,5.62)	2599	127	20565.12	6.18 (5.1,7.25)	1.223 (1.004,1.491)	0.05	1.095 (0.892,1.344)	0.39	
40–50	8587	785	66837.74	11.74 (10.92,12.57)	2114	227	15941.37	14.24 (12.39,16.09)	1.197 (1.033,1.386)	0.02	1.125 (0.969,1.306)	0.12	
> = 50	10742	1635	70632.14	23.15 (22.03,24.27)	2677	393	17049.69	23.05 (20.77,25.33)	0.981 (0.879,1.094)	0.73	0.971 (0.868,1.085)	0.60	
Comorbidity													0.51
0	38371	2183	308095.21	7.09 (6.79,7.38)	9173	540	72570.45	7.44 (6.81,8.07)	1.05 (0.956,1.154)	0.31	1.089 (0.989,1.2)	0.08	
> = 1	5444	844	32478.72	25.99 (24.23,27.74)	1781	271	10862.21	24.95 (21.98,27.92)	0.966 (0.844,1.106)	0.62	0.995 (0.868,1.14)	0.94	

^†^PY, person-years.

^‡^Incidence rate, per 1000 person-years.

^§^Multivariate analysis including age, gender, monthly income, Charlson’s comorbidity index, comorbidities (Hypertension, Hyperlipidemia, Gout, Polycystic ovaries, Gestational diabetes, Depression, Obesity, Chronic pancreatitis, Hepatitis B infection and Hepatitis C infection) and medications (Statins, Atypical Antipsychotics, HIV-drug, Corticosteroids for systemic, Immunosuppressants), where death were regarded as competing risks.

cHR: crude hazard ratio; aHR: adjusted hazard ratio; CI: confidence interval.

The incidence of type 2 DM stratified by age is shown in Table 3. Here we used the propensity score-based analysis to confirm the hazard ratio of type 2 DM among patients < 30 years of age. The multivariate regression analysis revealed that the aHR of type 2 DM was 2.45 in the appendectomy patients < 20 years (95% CI, 1.039–5.776) compared to controls. The propensity score-matching analysis revealed that the aHR of type 2 DM in the appendectomy patients < 20 years was 2.525 (95% CI, 1.116–5.695) compared to controls. The propensity score-based analysis revealed that the aHR of type 2 DM in the appendectomy patients < 30 years was 1.36 (95% CI, 1.02–1.813) compared to controls.

**Table 3:** Hazard ratio of incident diabetes mellitus stratified by age.

Stratify age	aHR^a^ (95% CI)	p-value^‡^	aHR^b^ (95% CI)	p-value^‡^	aHR^c^ (95% CI)	p-value^‡^
<20	2.45(1.039,5.776)	0.041	2.52(1.116,5.695)	0.026	2.449(1.085,5.528)	0.031
<30	**1.347(1.009,1.798)**	**0.043**	1.36(1.02,1.813)	0.036	1.369(1.027,1.824)	0.032
20–30	1.239(0.907,1.691)	0.178	1.26(0.924,1.717)	0.144	1.268(0.931,1.727)	0.132
30–40	1.071(0.875,1.31)	0.509	1.144(0.937,1.397)	0.188	1.147(0.939,1.401)	0.178

^a^ Adjusted for all variables listed in Table 1.

^b^Adjusted for propensity score.

^c^Adjusted for strata by quintiles based on propensity scores.

^‡^All analyses incorporated in regard to death as competing risks.

**Fig 2:**
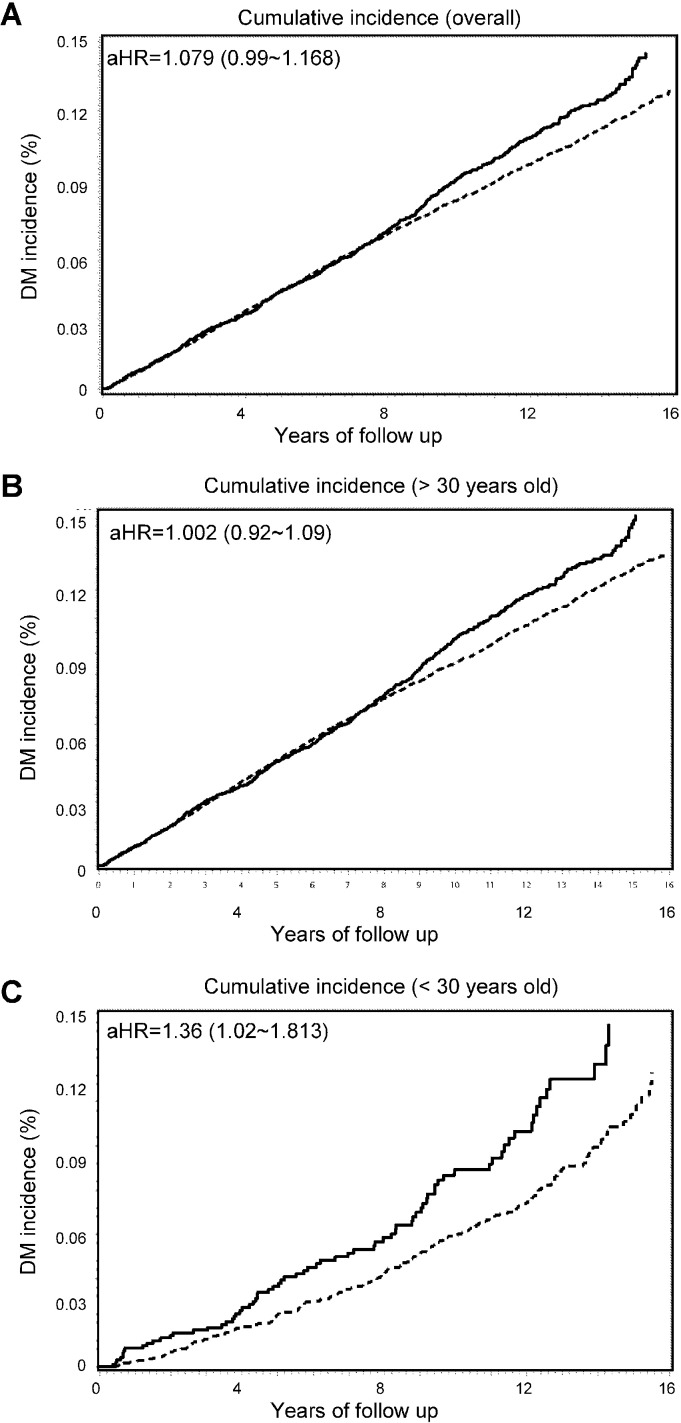
Cumulative incidence of diabetes mellitus (DM) between the appendectomy and comparison cohorts comparing the cumulative incidence of DM between the appendectomy and comparison cohorts among the overall patients and those patients younger or older than 30 years of age.

Moreover, the cumulative incidences are shown in Fig 2. The adjusted HR of type 2 DM in all patients with appendectomy was 1.079 (95% CI, 1.041–1.839), that in patients > 30 years of age with appendectomy was 1.002 (95% CI, 0.92–1.09), and that in patients < 30 years of age with appendectomy was 1.36 (95% CI, 1.02–1.813) compared to controls. The incidence of type 2 DM stratified by follow-up period post-appendectomy is shown in Table 4. The incidence of type 2 DM within 3 years post-appendectomy follow-up was higher in the appendectomy patients with an HR of 2.017 (P = 0.03). In contrast, the incidence of type 2 DM > 10 years post-appendectomy follow-up was lower in the appendectomy group with an HR of 1.442 (P = 0.223).

**Table 4:** Hazard ratio of incident diabetes mellitus in patient at aged<30 year during post appendectomy follow-up.

post appendectomy follow time (year)	aHR^a^ (95% CI)	p-value^‡^	aHR^b^ (95% CI)	p-value^‡^	aHR^c^ (95% CI)	p-value^‡^
**<3**	**2.017(1.07,3.802)**	**0.03**	**1.946(1.048,3.612)**	**0.035**	**1.951(1.049,3.629)**	**0.035**
**3–7**	**1.764(1.052,2.957)**	**0.031**	**1.861(1.123,3.084)**	**0.016**	**1.786(1.076,2.962)**	**0.025**
<7	1.705(1.146,2.538)	0.009	1.732(1.17,2.563)	0.006	1.684(1.138,2.493)	0.009
7–10	1.232(0.659,2.303)	0.513	1.223(0.668,2.24)	0.514	1.242(0.679,2.271)	0.482
> = 10	1.442(0.8,2.599)	0.223	1.382(0.771,2.478)	0.278	1.42(0.792,2.547)	0.239

^a^ Adjusted for all variables listed in Table 1.

^b^Adjusted for propensity score.

^c^Adjusted for strata by quintiles based on propensity scores.

^‡^All analyses incorporated in regard to death as competing risks.

## Discussion

In this large retrospective population-based cohort study, we found no statistically significant association between appendectomy and type 2 DM in the overall population (aHR, 1.079; 95% CI, 0.997–1.168). However, appendectomy increased the risk of type 2 DM among patients < 20 years (aHR, 2.45; 95% CI, 1.039–5.776) and those <30 years (aHR, 1.347; 95% CI, 1.009–1.798). In the random frequency-matched cohort, all confounding factors except Charlson Comorbidity Index score and number of visiting clinics were equally distributed in the appendectomy and control groups. To reduce selection bias, a multivariate regression analysis was conducted of a propensity score-matched cohort, which revealed that the adjusted HR of type 2 DM in the appendectomy patients < 20 years and those < 30 years were 2.52 (95% CI, 1.116–5.695) and 1.36 (95% CI, 1.02–1.813) compared to controls. Since the number of appendectomy patients < 20 years of age was limited, we may have overestimated the impact of age on appendectomy and the development of type 2 DM. Therefore, we concluded that appendectomy prior to 30 years of age is an independent risk factor for the development of type 2 DM.

Apart from significant results in the age subgroup analyses confirmed by the different statistical methods, our results were adjusted by many confounding factors, including hypertension, hyperlipidemia, gout, polycystic ovaries, depression, GDM, depression, obesity, chronic pancreatitis, HBV, HCV, statins, atypical antipsychotics, HIV drugs, corticosteroids, and immunosuppressants. This implies that age indeed had a profound effect on the association between appendectomy and type 2 DM. On the other hand, we found that sex did not influence the association between appendectomy and type 2 DM (P_interaction_ = 0.88). The aHR of type 2 DM incidence in the male appendectomy group was 1.058 (P = 0.34), while that in the female appendectomy group was 1.072 (P = 0.20) compared to controls. Furthermore, the major outcome was a new diagnosis of DM (ICD-9 250.0–250.9), which included type 1 and 2 DM. Because patients with a history of DM before the index year and subjects < 18 years were excluded, we believe that most of newly diagnosed DM patients after appendectomy have type 2 DM.

Appendicitis is one of the most common acute abdominal diagnoses and mainly results from bacterial infection rather than obstructions within the organ [22]. Appendicitis is a clinical emergency for which surgery remains the gold standard of treatment. Similar to our results, appendectomy is associated with various human diseases [16–21]; however, the mechanism of their associations is largely unknown. Some possible explanations might address their correlations. First, the appendix is considered a safe house to shelter the commensal intestinal flora through biofilm formation. Second, the appendix is considered part of the immune system because of its abundant gut-associated lymphoid tissue. As a result, appendectomy and prophylactic antibiotics given before appendectomy surgery may disrupt gut microbiota configurations, subsequently supporting diabetes development. Interestingly, several population-based studies from Taiwan were conducted based on the National Health Insurance Research Database (NHIRD) [16, 18–20]. The NHIRD provided the opportunity to investigate the associated with subsequent disease development and appendectomy in real-world settings in a large population sample in Taiwan and can help accumulate evidence for the creation of appropriate surgical interventions to remove the appendix since it might cause long-term health effects.

A recent study revealed that the human appendix accommodates a wealth of microbiota different from other niches and shows substantial diversity [23]. Another study suggested that appendiceal dysbiosis appears in insulin resistance morbidly obese (IR-MO) patients, with a reduction of butyrate-producing bacteria that are necessary to maintain gut integrity together with an increase in mucin-degrading bacteria and opportunistic pathogens [24]. Meadows identified that *Firmicutes* are associated with weight gain and insulin resistance because they supply extra energy [25]. Verdam et al. described that an increase in *Firmicutes* and a decrease in *Bacteriodetes* is linked to the presence of obesity and inflammation [26]. Indeed, the appendiceal microbiota have been recognized as an innovate actor capable of modulating the host metabolism through building the appendiceal immune function [24].

Most importantly, our study showed that appendectomy increases the risk of type 2 DM, particularly when performed in patients before middle age. We believe that dysbiosis or imbalance of the enteric microbiota may bolster chronic inflammation through intestinal barrier breakdown, microbiota outgrowth, and translocation of the microbiota. Moreover, immunity may weaken with age, thereby lessening inflammation. This might address the possibility of the impact of age on appendectomy to the emergence of type 2 DM. Furthermore, other risk factors may attenuate the effect of appendectomy on the development of diabetes after middle age. Appendectomy appeared to be associated with the risk of diabetes development approximately 3–7 years later. We think that changes in the gut microbiota composition could be prominent at the early stage post-appendectomy due to bacterial infection, surgery, and antibiotic use. However, dysbiosis of the enteric microbiota could be recovered by a degree, so animal studies should be performed to clarify the association between appendectomy and type 2 DM.

### Study limitations

There are some limitations in our study. First, lifestyle data regarding dietary habits, exercise, smoking, socioeconomic status, and hereditary background were not available in this retrospective cohort study. Second, all comorbidities were diagnosed using ICD-9 codes, the accuracy of which should be discussed. Third, we did not separate perforated appendicitis from non-perforated appendicitis or distinguish between laparoscopic appendectomy and open appendectomy. Fourth, despite of our thorough study design and adjustment for confounding factors, some selection bias may have resulted from the retrospective nature of this study.

## Conclusions

In conclusion, appendectomy performed in patients < 30 years is associated with the risk of type 2 DM independent of hypertension, hyperlipidemia, gout, polycystic ovaries, depression, GDM, depression, obesity, chronic pancreatitis, HBV, HCV, statins, atypical antipsychotics, HIV drugs, corticosteroids, and immunosuppressants. The results of this study implicate that the appendix protects against type 2 DM development before middle age. Further studies should be performed to clarify the role of appendectomy in the development of diabetes.

## Supporting information

S1 Table Incidence and hazard ratios of diabetes mellitus for appendectomy patients compared with non-appendectomy cohort by demographic characteristics and comorbidities. (DOCX)
