# Supplementary material for: Impact of age at appendectomy on development of type 2 diabetes: A population-based cohort study
Source: PLoS One. 2018 Oct 16;13(10):e0205502. doi: 10.1371/journal.pone.0205502 (PMC6191136; doi:10.1371/journal.pone.0205502)
Supplement: S1 Table — (DOCX) [file pone.0205502.s001.docx]

Supplemental Table 5: Incidence and hazard ratios of diabetes mellitus for appendectomy patients compared with non-appendectomy cohort by demographic characteristics and comorbidities:

|  | Subjects with non-appendectomy | | Subjects with appendectomy | | Appendectomy cohort vs. Non- appendectomy cohort | | | |
| --- | --- | --- | --- | --- | --- | --- | --- | --- |
|  | event | Incidence^‡^ (95% CI) | event | Incidence^‡^ (95% CI) | cHR (95% CI) | p-value | aHR^§^(95% CI) | p-value |
| **DM diagnosis was defined by stringent criteria** | | |  |  |  |  |  |  |
| All patients | 2358/43815 | 6.92 (6.64–7.2) | 618/10954 | 7.41 (6.82–7.99) | 1.066(0.976,1.165) | 0.1552 | 1.067(0.974,1.168) | 0.1623 |
| Age<30 | 120/14041 | 1.00 (0.82–1.18) | 43/3564 | 1.44 (1.01–1.87) | 1.457(1.029,2.064) | 0.0341 | 1.436(1.013,2.035) | 0.0422 |
| **DM was diagnosed by specialist of family medicine or endocrinology** | | | | |  |  |  |  |
| All patients | 2460/43815 | 7.22 (6.94–7.51) | 655/10954 | 7.85 (7.25–8.45) | 1.066(0.976,1.165) | 0.1552 | 1.08(0.989,1.18) | 0.0865 |
| Age<30 | 137/14041 | 1.14 (0.95–1.34) | 48/3564 | 1.61 (1.15–2.06) | 1.42(1.022,1.972) | 0.0365 | 1.415(1.017,1.969) | 0.0395 |

^†^PY, person-years. ^‡^Incidence rate, per 1000 person-years.

^§^Multivariate analysis including age, gender, , monthly income, Charlson’s comorbidity index, comorbidities (Hypertension, Hyperlipidemia, Gout, Polycystic ovaries, Gestational diabetes, Depression, Obesity, Chronic pancreatitis, Hepatitis B infection and Hepatitis C infection) and medications (Statins, Atypical Antipsychotics, HIV-drug, Corticosteroids for systemic, Immunosuppressants), where death were regarded as competing risks.

cHR: crude hazard ratio; aHR: adjusted hazard ratio; CI: confidence interval.
